# Reliability of isometric subtalar pronator and supinator strength testing

**DOI:** 10.1186/s13047-015-0075-8

**Published:** 2015-04-17

**Authors:** Marco Hagen, Matthias Lahner, Martin Winhuysen, Christian Maiwald

**Affiliations:** Biomechanics Laboratory, Institute of Sport and Movement Sciences, University of Duisburg-Essen, Gladbeckerstraße 182, Essen, 45141 Germany; Clinics for Orthopaedic and Trauma Surgery, University Hospital St. Joseph, Ruhr-University Bochum, Gudrunstraße 56, Bochum, 44791 Germany; Department of Applied Human Movement Science, Technische Universität Chemnitz, Chemnitz, 09107 Germany

**Keywords:** Isometric muscle strength, Subtalar joint, Pronation, Supination, Limits of agreement, Minimum detectable change

## Abstract

**Background:**

Due to the specific anatomy of the subtalar joint with its oblique axis, isometric pronator and supinator strength is not well documented. The purpose of this study was to determine intra- and between-session reliability of pronator and supinator strength and lower leg muscle activity measurements during maximum voluntary isometric contractions (MVIC).

**Methods:**

Pronator and supinator peak torques (PT), with and without supplementary visual muscle strength biofeedback (FB), and muscular activities of peroneus longus (PL) and tibialis anterior (TA) were assessed twice 3 days apart by the same examiner in 21 healthy young male adults (mean age: 27.6 years; SD = 3.9). Limits of agreement (LoA) and minimum detectable change (MDC) were evaluated.

**Results:**

By applying FB, reliability of both pronator and supinator PT was improved: LoA were reduced from 32% to 26% and from 20% to 18% and MDC from 20% to 15% and from 16% to 12% in supinator and pronator PT, respectively. Learning effects in pronator and supinator PT (p < 0.05), which were present without FB, were eliminated using FB. Except for TA during pronation, muscle activities showed low reliability indicated by LoA of 51% to 79%.

**Conclusions:**

Using supplementary biofeedback, isometric subtalar pronator and supinator strength testing is reliable in healthy subjects. LoA of 18% and 26% have to be exceeded for pronator and supinator PT, respectively, to detect relevant effects in repeated measures.

## Background

The muscle strength of the subtalar pronators and supinators plays a key role in medio-lateral foot stability. To prevent recurrent ankle sprains, subtalar joint-specific pronator strength training is recommended to counteract peroneal muscle weakness [1,2] and to enhance pronator-to-supinator strength-ratio [3,4]. Strengthening the supinators increases the eccentric contraction capacity of the muscles of the deep posterior compartment (tibialis posterior, flexor hallucis longus and flexor digitorum longus muscles) [5]. This is potentially beneficial in the prevention of running-related overuse injuries, like exercise related lower leg pain [6,7], patellofemoral pain syndrome [8], patellar tendinopathy [9], achillodynia [10-12] and plantar fasciitis [13,14].

Previous research on subtalar strength testing is based on the peak torques (PT) generated during isokinetic measurements [15-21]. Although isokinetic strength testing of the pronators and supinators has shown high day-to-day correlations, its use in assessing maximum voluntary strength is limited. For instance, accelerative effects that occur when the movement direction is changed [22] confound PT. Furthermore, the joint angle, at which PT occurs, depends on the chosen velocity [23]. Therefore, maximum voluntary isometric contractions (MVIC) are recommended, as the resulting isometric voluntary muscle strength reflects the real muscle capacity [24]. Moreover, MVIC measurements are advantageous when surface electromyographic (EMG) techniques are used, because there is less displacement of muscle fibres underneath the surface electrodes compared to dynamic movements [25].

Measurement of MVIC requires maximum activation of all motor units that innervate the target muscles with respect to the strength task. Variations in MVIC outcomes between different trials of strength testing are based on fluctuations in maximum neural activity of both agonistic and antagonistic muscles [26]. Such an error in strength testing can be attributed to motivation, for instance [27-29]. Therefore, subjects are usually verbally encouraged by the tester to achieve the highest MVIC output possible [30].

Another factor that might influence the strength testing outcome, which is not well documented in the literature, is the precision required to perform the motor task. This problem arises when the MVIC recording also requires an accurate direction of joint movement. This phenomenon is particularly present in the triplanar axis of the subtalar joint. To address this, we developed a functional pronator and supinator strength training and testing machine with a driveshaft aligned with the subtalar joint axis as defined by Isman and Inman [31]. The strength testing apparatus was equipped with two one-dimensional force transducers which were connected to the driveshaft [32]. Thus, we were able to detect the resulting pronator and supinator torque within the subtalar joint-specific movement plane. To register the real exertion of the pronators and supinators, our force-transducer-arrangement required a precisely coordinated triplanar movement of the foot around the subtalar joint axis. If, however, a subject performed an inaccurate pronation or supination out of the intended direction during strength testing, the force transducer only registered a portion of the strength performance of the target muscles. Therefore, during MVIC measurement a biofeedback method was applied while the signal of the force transducer was displayed on the monitor. As suggested by James and Graves [33], supplementary FB helps to increase PT especially during unfamiliar contractions. We assumed that providing FB could enhance the reliability of pronator and supinator MVIC testing, as subjects would perform a more accurate triplanar subtalar motion and, consequently, come closer to approaching their individual strength capacity.

To analyze muscle strength performance of the mediolateral prime movers of the foot based on a valid and reliable method, the purpose of this study was to investigate the day-to-day reliability of muscle strength and myoelectric activity when isometric functional anatomical pronator and supinator MVIC tests were performed. Furthermore, it was hypothesized that administering supplementary visual feedback of the force signal would enhance the precision of the required motor task and, subsequently, the reliability of strength testing within the subtalar-joint-specific movement plane.

## Methods

### Study design

On two separate days, maximum isometric strength tests of the pronators and supinators of the dominant foot were administered to 21 male volunteers ranging from 21 to 38 years (see Table 1 for anthropometrics). The subjects were sport students at the local university. None of the participants reported having any contraindications to resistive exercise or major neuromusculoskeletal dysfunction of the lower extremities in the past two years. Background information on the experimental procedures was provided to the participants, and informed written consent was collected prior the first test session. The study was approved by the ethics committee of the local university hospital in accordance with the Helsinki Declaration.

**Table 1:** **Anthropometric data**

	**Mean**	**SD**	**Min**	**Max**
Age (years)	24.8	2.7	20	31
Height (m)	1.81	0.08	1.68	1.93
Mass (kg)	79.5	6.8	61.4	90
Body-Mass-Index (kg/m^2^)	23.9	1.8	21.8	27.7
Foot length (cm)	27.6	0.9	26.5	29.0

### Strength testing

**Figure 1:**
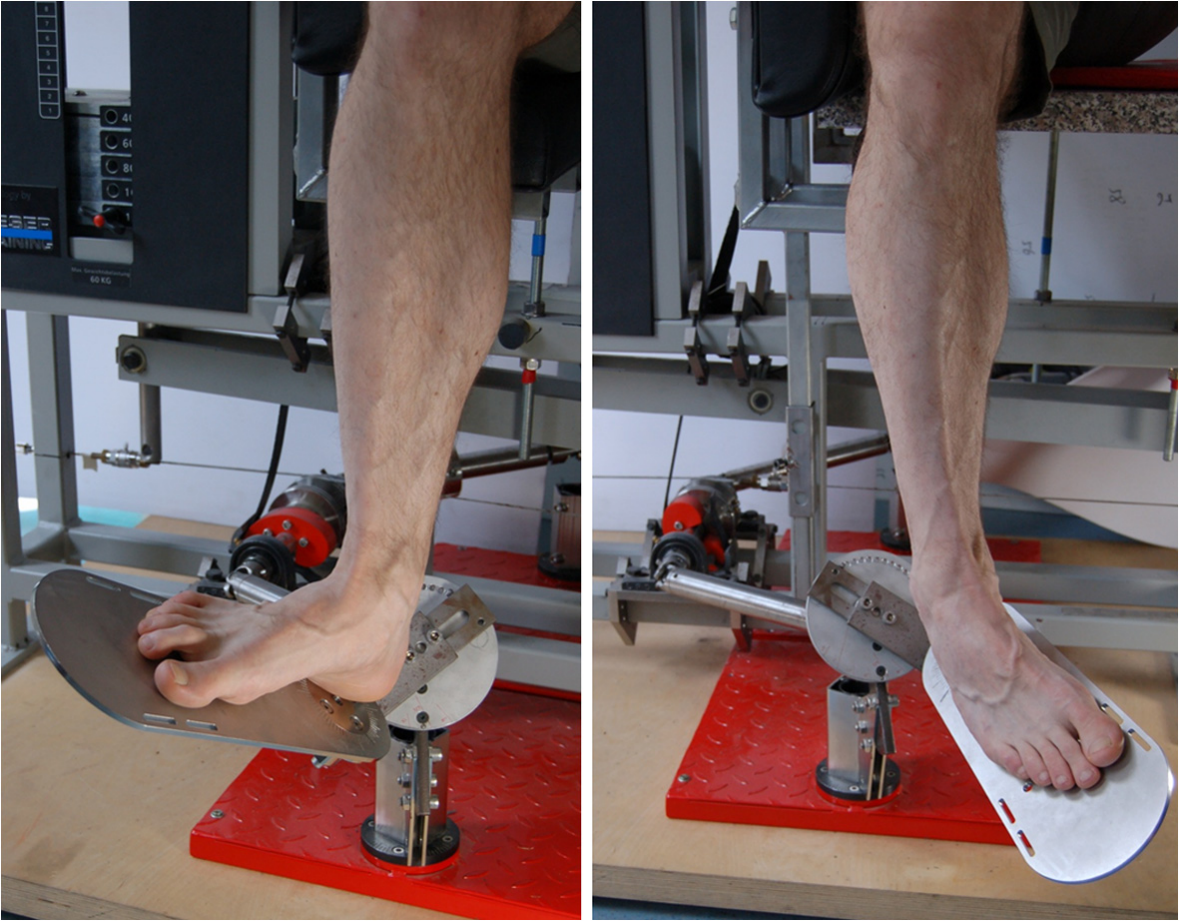
Machine-based subtalar joint-specific pronation (left) and supination (right).

**Figure 2:**
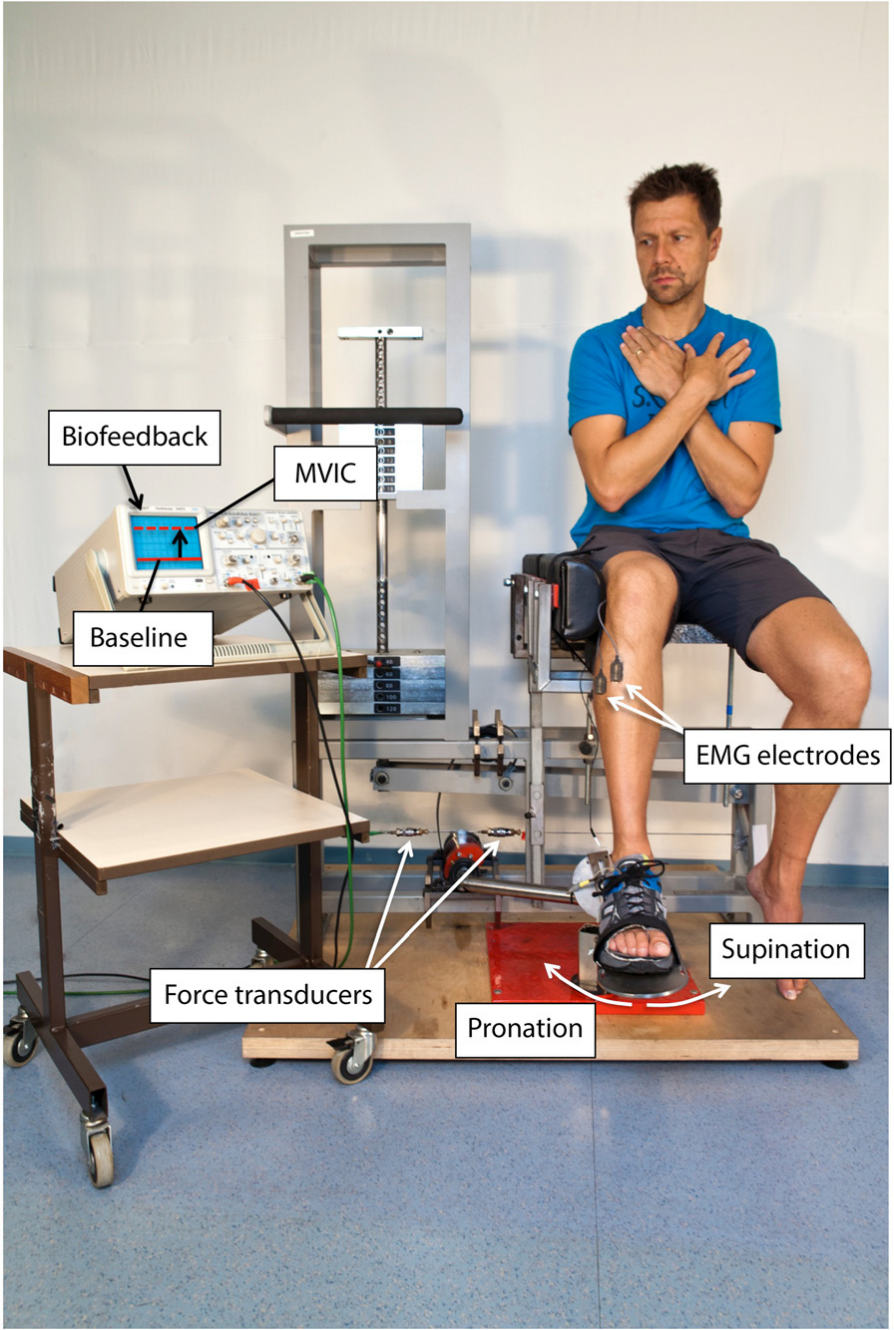
Setup.

A specific foot apparatus adjustable in all three dimensions was constructed to perform pronator and supinator MVIC testing (Figure 1). The movement axis was oriented parallel to the subtalar joint axis, which deviates about 23° medially and about 41° dorsally from the longitudinal foot axis [31]. The foot apparatus was connected via a cardan driveshaft and a pull rope to the adjustable weight block and could also be used as a training machine. A sport shoe (size: US 10) was mounted onto the foot plate. The tip of the shoe’s upper was cut off so that the machine could be used by subjects with variable foot lengths between 26.5 cm to 29.5 cm (i.e. shoe sizes US 8.5 to 11.5). In neutral position, with the lower leg perpendicular to sole of the foot, the foot plate of the apparatus was aligned parallel to the floor and to the longitudinal axis of the foot. During testing, the forefoot was secured additionally using a belt. MVIC testing was performed in a seated position with hip and knee joints each positioned at approximately 90 degrees. This eliminated the mechanical influence of the gastrocnemius muscle on the range of ankle and subtalar joint motion and the resulting pronator and supinator strength outcome. Associated hip and knee motions were prevented using straps which were placed around the thigh (Figure 2).

During isometric strength measurements, the apparatus was constrained by two steel wires which connected the driven shaft to the machine frame. Subsequently, two Kistler force transducers (Kistler, Winterthur, CH) placed in the steel wires were used to measure the maximum resulting voluntary isometric pronator and supinator torques. These data were then used to determine pronator and supinator PT. Each subject performed three trials of maximum voluntary isometric pronations and supinations (MVIC) with their foot in neutral position in two test conditions in random order: a) Regular strength testing with verbal encouragement by the tester, and b) strength testing identical to a) plus additional biofeedback (FB) of the force sensor. For the purpose of biofeedback, the real time signal of the force sensor was displayed on a monitor. In condition b), the subjects were verbally instructed “to force the signal to the border of the screen”. For both tests, participants were instructed to perform ramp contractions and to hold MVIC for at least two seconds. The maximum of each torque-time curve was registered as pronator and supinator PT, respectively. A two minute rest was permitted between the trials to prevent fatigue build-up [30].

### Plantar pressure measurement

Plantar pressures during the MVIC trials were recorded by a capacitive pressure insole (Pedar, Novel, Munich, 99 sensors), which was inserted into the shoe. In a pilot test, we observed that the point of force application during supinator MVIC was systematically located under the lateral forefoot, and during pronator MVIC under the medial forefoot. Peak forefoot pressures of the 52 distal sensors of the insole were sampled at a frequency of 50 Hz. The sensor arrangement was divided into the two regions of interest: medial and lateral forefoot. Peak medial pressures (PP med FF) and peak lateral pressures (PP lat FF) under the forefoot were recorded during pronation and supination MVIC, respectively. Data recording and inspecting was conducted using standard Novel software package products (Novel Tools V13.3.30 and Novel Multimask V13.3.30, Novel GmbH, Munich, Germany).

### EMG

Myoelectric activity of the tibialis anterior (TA) and the peroneus longus (PL) were recorded simultaneously by surface EMG electrodes with an interelectrode distance of 10 mm (DE 2.1, Delsys, Boston, USA). After careful preparation of the skin by shaving and abrasion with sandpaper, the electrodes were placed on the muscle bellies according to SENIAM recommendations [34] by the same investigator (MW). The correct placements were verified by manual tests and voluntary contractions. The reference electrode was placed over the lateral malleolus. All electrode placements were marked with water resistant ink, and the subjects were asked to redraw the marks if necessary after personal hygiene. Thus, reproducible electrode positioning could be ensured.

Integrated EMG (IEMG) was recorded over a period of one second during each MVIC trial. To compare the amount of activity between session 1 and 2, IEMG was normalized by using daily life reference tasks: 1. Heel raise while keeping the knee fully extended for PL, and 2. toe raise for TA. We assumed that performing these tasks would not cause learning effects between session 1 and session 2. Heel and toe raise tasks were performed unilaterally. Both heel and toe raise contractions were done according to De Luca [35], who recommends isometric normalization procedures below 80% of MVIC. Subjects were instructed to execute maximum voluntary dorsal and plantar flexion for at least five seconds, respectively. While doing this, participants were allowed to touch the wall with index and middle fingers to stabilize their standing posture. During an experimental session lasting ~ 90 minutes, there was an increasing risk of varying physiological properties of skin, subcutaneous tissue and muscle due to sweat and changes of temperature [36]. To avoid these confounding factors which would have interfered with the conductivity of the skin-electrode-interface [35], two normalization trials were recorded before and after the test procedure, respectively. Correspondingly, IEMG was recorded over a period of one second during the isometric normalization contraction. The mean of the four trials (two before and two after strength testing) was evaluated as a reference. For the pronation and supination MVIC measurements, IEMG of PL and TA in % of this reference were evaluated.

EMG and force signals were A/D converted (NI-DAQPad-6015, National Instruments, Texas, USA) and sampled with 4000 Hz with a 12 bit resolution. Raw EMG signal was filtered (bandpass 10–1000 Hz) and full-wave rectified. All data were recorded using custom written software in LabView (National Instruments, USA). During the subsequent process of analysis, all EMG signals were smoothed using a Butterworth 4th order low pass filter with a cut-off frequency of 10 Hz.

Approximately one week before the experiment started, subjects were familiarized with the setup. After verbal explanation and a practical demonstration, participants performed a number of practice strength tests. All instruction procedures were repeated on the testing days. Then subjects were prepared for measurement and EMG electrodes were glued to the dominant leg. Leg dominance was determined as the leg which is preferred for kicking a ball. Participants conducted a 10-minute warm-up on a bicycle ergometer before EMG-normalization procedures and MVIC testing. At least 72 hours after the first test session, the experimental procedure was repeated at the same time of day (± one hour). All experimental applications were conducted by the same tester (MW).

### Statistical analysis

Three valid trials were used to calculate all measures of reliability. All data were monitored for heteroscedasticity by calculating the Pearson product–moment correlation (Het_R) between intrasubject standard deviation of repeated measures and their mean value. Het_R ≥ 0.7 (Het_R^2^ ≥ 0.5) was defined as the threshold for classifying a variable as heteroscedastic [37]. Since all analyzed datasets were homoscedastic, the reliability measures were calculated from the raw variables.

For assessing intra-session reliability, the root mean square error (RMSE) was calculated, which quantifies the precision of individual scores on a test [38]. RMSE is represented by the square root of the error mean square of the interaction factor of a two-factor repeated measures ANOVA (here: trials x test sessions). The factor *trial* includes the three MVIC repetitions within one day, and the factor *session* includes the repetition of the entire lab session on the two separate days [39]. RMSE is given in the unit of the variable and can therefore be directly related to the parameter of interest. To calculate the minimum detectable change (MDC) which is needed to identify clinically relevant effects between repeated measures of one subject, RMSE has to be multiplied by a factor of 2.77 [40]. In other words, 95% of repeated measurements on the same subject lie within the interval of ± 2.77 x RMSE. MDC is also expressed as a percentage (MDC/mean of all observations x 100) [41].

Bland and Altman plots were used to evaluate the between-session reliability. Bias (mean difference between the two test sessions) and the limits of agreement (LoA) as the random error component (1.96 x standard deviation of the difference between the two test sessions) were calculated [42].

As strength testing is usually applied to identify the effects of an intervention, and a 20–30% increase in strength of the lower extremities was reported in healthy men after a period of strength training [43,44], the following thresholds for the percentaged LoAs and MDCs were determined: ≤ 20%: good reliability, 20–30%: acceptable reliability, > 30–40%: poor reliability, and > 40%: unacceptable reliability.

Additionally, paired t-tests were conducted to identify learning effects from session 1 to session 2. The level of significance was set at α = 0.05. The statistical analyses were performed using Microsoft Office Excel 2010 (Microsoft, Redmond, USA) and SPSS 18.0 (IBM, Chicago, USA).

### Statement of institutional review board approval

The study was approved by the ethics committee of the Faculty of Medicine of the University of Duisburg-Essen in accordance with the Helsinki Declaration. Approval No.: 11-4887-BO.

## Results

The reliability parameters (LoA, MDC) are presented in Table 2 according to peak torques, peak plantar pressures and the IEMGs of TA and PL.

**Table 2:** **Reliability scores of isometric pronator and supinator muscle strength, peak plantar pressures and muscle activities**

	**Day 1**			**Day 2**			**Reliability**						**Learning effects**
	**Mean 1**	**SD 1**	**Het_R** ^**2**^	**Mean 2**	**SD 2**	**Het_R** ^**2**^	**RMSE**	**MDC (abs)**	**MDC %**	**Bias (abs)**	**LoA (abs)**	**LoA (%)**	***t*** **-test (P-Value)**
**Pronation (no FB)**													
Pronator PT (Nm)	17.5	2.7	0.22	18.5	2.6	0.06	1.0	2.9	16.2	(-0.9)	3.5	19.6	<0.05
PP med FF (kPa)	54.3	16.1	0.22	59.2	15.5	0.00	6.6	18.2	32.3	(-4.9)	23.9	42.1	0.17
IEMG PL (%)	134.9	52.8	0.14	129.5	48.4	0.13	14.4	39.9	30.3	41.4	81.1	61.4	0.64
IEMG TA (%)	114.0	27.3	0.18	111.3	26.7	0.11	7.5	20.9	18.5	2.7	19.1	33.2	0.59
**Pronation (FB)**													
Pronator PT (Nm)	19.0	2.4	0.00	19.1	2.4	0.06	0.8	2.3	11.9	(-0.1)	3.4	17.7	0.76
PP med FF (kPa)	59.0	18.3	0.03	62.3	21.5	0.12	6.1	17.0	28.1	(-3.3)	21.6	35.6	0.30
IEMG PL (%)	130.4	41.6	0.13	129.6	48.4	0.13	13.6	37.6	28.9	0.8	65.7	50.5	0.93
IEMG TA (%)	108.0	21.7	0.00	105.0	20.0	0.00	6.8	18.9	17.7	3.0	19.1	17.9	0.25
**Supination (no FB)**													
Supinator PT (Nm)	13.3	5.7	0.07	14.8	6.1	0.12	1.0	2.81	20.0	2.4	4.6	32.9	<0.05
PP lat. FF (kPa)	103.3	39.7	0.03	109.1	31.8	0.02	10.2	28.2	26.6	(-5.5)	34.2	32.2	0.22
IEMG PL (%)	80.0	54.7	0.45	75.5	34.7	0.32	10.9	30.1	38.7	0.8	61.5	79.1	0.68
IEMG TA (%)	39.0	25.3	0.46	37.9	20.3	0.26	5.5	15.2	39.6	0.6	27.5	71.6	0.99
**Supination (FB)**													
Supinator PT (Nm)	14.7	5.5	0.22	15.4	5.6	0.03	0.8	2.2	14.9	(-0.6)	3.9	26.0	0.17
PP lat. FF (kPa)	126.5	33.7	0.02	128.0	30.9	0.00	9.0	25.0	19.6	(-1.6)	17.7	27.3	0.74
IEMG PL (%)	82.6	41.6	0.17	83.3	40.3	0.26	11.2	31.0	37.4	24.2	47.4	57.1	0.91
IEMG TA (%)	32.9	22.6	0.44	32.8	17.2	0.27	3.9	10.9	32.9	(-0.1)	22.1	67.2	0.97

Legend: SD, standard deviation; MDC, minimum detectable change; MDC %, minimum detectable change as a percentage of session means; LoA, limits of agreement; Het_R^2^, Index of heteroscedasticity, FB, feedback; PT, peak torque; PP med. /lat. FF, peak plantar pressure under medial/lateral forefoot; IEMG, integrated EMG; PL, peroneus longus muscle; TA, tibialis anterior muscle.

In the non-FB conditions, the participants exhibited good inter-session reliability only for pronator PT (LoA: 19.6%). All other parameters of interest did not reach LoA-values below 30%. In the non FB-conditions, MDCs revealed good intra-session reliability for pronator PT, supinator PT and TA during pronation. During non-FB pronation PP showed acceptable intra-session reliability.

**Figure 3:**
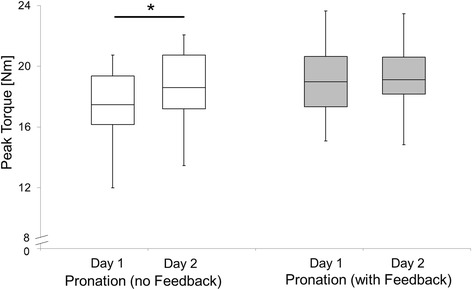
Boxplots of pronator peak torques in sessions 1 and 2 with (grey) and without FB (white). *(p < MS: 1590423483128936 - Reliability of isometric subtalar pronator and supinator strength testing0.05) = Significant learning effect between sessions 1 and 2. Boxes indicate median, lower and upper quartile; whiskers extend to the minimum and maximum.

**Figure 4:**
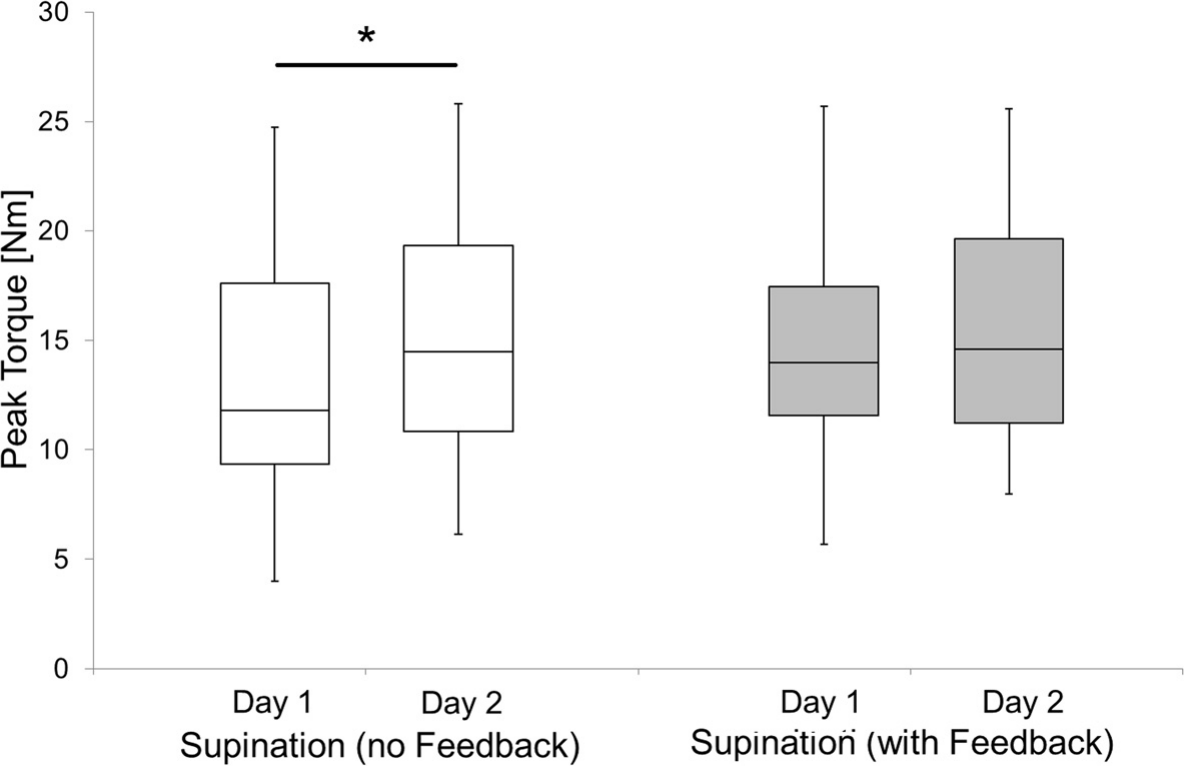
Boxplots of supinator peak torques in sessions 1 and 2 with (grey) and without FB (white). *(p < 0.05) = Significant learning effect between session 1 and 2. Boxes indicate median, lower and upper quartile; whiskers extend to the minimum and maximum.

In general, the participants exhibited good and acceptable reliability for pronator and supinator PT, respectively, when strength testing was supplemented by FB. Compared to non-FB conditions, both pronator (+5%) and supinator PTs (+7%) improved with the use of FB and were accompanied by reduced MDCs of 4.3% and 5.1% in pronator and supinator PT, respectively. Supplementary FB also increased intersession reliability, indicated by the relative LoA of 2% and 7% in pronator and supinator PT, respectively. In contrast to FB conditions, systematic bias between test sessions 1 and 2 was detected in pronator (+5%; p < 0.05) and supinator PT (+9%; p < 0.05) in the non-FB conditions (Figures 3 and 4).

Increasing plantar pressures of 7% and 20% under the medial (pronator MVIC) and lateral midfoot (pronator MVIC), respectively were accompanied by reduced MDCs (pronation: minus 4%; supination: minus 7%) and LoA (pronation: minus 6%; supination: minus 5%) when FB was given.

Compared to the muscle strength parameters, muscle activities (IEMGs) were less reliable. The lowest MDCs of 17.7% and 18.5% were found in TA during pronation with and without FB, respectively. During pronation, relative LoA of TA decreased from 33.2% to 17.9% after providing FB. Despite providing supplementary FB, LoA-values above 50% across all strength measurements revealed unacceptable inter-session reliability of PL muscle activities.

## Discussion

The purpose of the present study was to investigate the reliability of isometric subtalar joint-specific lower leg muscle strength and neuromuscular activation testing with and without providing visual FB of the force signal. The principal finding of our experiment was that isometric strength measurements result in good to acceptable reliability in FB conditions. The minimum detectable change (MDC), which represents the intra-session measurement error, was 12% in pronator PT and 15% in supinator PT. The limits of agreement (LoA), which reflect a non-random change in isometric muscle strength between the sessions, were 20% in pronator PT and 26% in supinator PT in the FB-conditions. As hypothesized, both MDC and LoA were reduced in pronator and supinator PT when strength testing was supplemented by visual FB.

The present findings are consistent with those of other studies that found an increase of muscle strength output by providing biofeedback [45,46] and knowledge of the results [47,48]. Our results are in agreement with those of Graves and James [33] who suggest providing biofeedback to obtain maximum isometric muscle strength during unfamiliar movements. Furthermore, our findings confirm the hypothesis that using biofeedback leads to reduced measurement errors according to the mechanical outcome measures, PT and peak plantar pressures. On the one hand, supplementary feedback can result in higher motivation to force the displayed MVIC signal to an even higher level on the monitor, meaning subjects come closer to approaching their individual strength capacity and enhancing the reliability in outcome measures [45]. On the other hand, participants get direct information about how accurate the muscle strength is applied with respect to the movement task. Both pronator and supinator MVIC measurements also favour the use of biofeedback in terms of eliminating learning effects from session 1 to session 2. In general, pronator PT demonstrated lower MDC- and LoA-scores compared with supinator PT. However, “pulling the foot upward and sideways” was more comprehensible for the subjects, possibly because it is more common in daily life.

Although we also found increasing reliability in most muscle activities in the biofeedback conditions compared to the non-biofeedback conditions, IEMG data of the lower leg muscles were less reliable compared to the strength parameters. Only muscle activities of TA during pronator MVIC recordings showed small intra-session measurement error with MDCs below 20%. By using supplementary FB, reduced LoA of about 18% were found for TA during pronation. Reduced reliability was found in TA and PL when activated to a lesser extent during supination compared to the pronation task. It can be concluded that the less these muscles are activated, the more noise-induced errors confound the IEMG outcome during the one-second period which was evaluated [25]. While intra-session reliability (MDC) was in the acceptable range of 18–30% during pronation, there were substantial measurement errors (LoA) between sessions, except for TA during pronation. The measurement errors ranged from a minimum of 51% for PL during pronation with FB to a maximum of 79% for PL during supination without FB. These results are consistent with Murley et al. [49], who found unacceptably large measurement errors between sessions for both intramuscular and surface-EMG recordings of selected lower leg muscles. Although the activities of all agonistic muscles showed less measurement error in the biofeedback conditions, their poor reliability leads us to question the use of lower leg surface EMG in MVIC testing in repeated measurements, even if an optimized biomechanical approach is used. It must be considered whether this level of error is acceptable for identifying e.g. neuronal training effects.

In the present study, an ad-hoc sample of healthy, young, male sports students was investigated. It may be speculated that a sample of female participants would have achieved lower reliability in muscle strength, as previous studies reported that women’s strength outcomes are generally more variable compared to men [50,51]. Higher variability could be also expected for strength testing outcomes in older populations because of less daily activity and, therefore, less proprioception of the ankle joint complex [52]. However, these speculations should be investigated in future studies. Another limitation of our study is that, due to ethical reasons, we were not able to measure the muscle activity of the deep posterior compartment, i.e. tibialis posterior, flexor hallucis longus and flexor digitorum longus muscles. These muscles have been suggested to be the main medial stabilizers of the foot with respect to the subtalar joint axis [31]. Their neuromuscular activation can only be recorded by applying invasive EMG techniques. Thus, assessing the neuromuscular contribution of the deep retrotibial muscles to subtalar joint-specific supination as performed in the present study remains a challenge for future research.

One further limitation is that our strength testing device has the same axis position for all participants. It should be acknowledged that variations in the spatial orientation of the subtalar joint axis and other foot axes have been found both within the population and in dynamic movements [53].

The results of the present study lead to important recommendations for testing pronator and supinator MVIC. The oblique subtalar joint axis makes reproducible isometric strength testing difficult, especially for determining supinator PT. Supplementary visual biofeedback helps to generate the highest possible isometric strength output [45]. As suggested by Annett [54], biofeedback has been considered in informational and motivational terms, whereas the motivational effect is simply a realization of the informative content of the feedback. Therefore, it can be concluded that the external feedback loop informing about the direction of the specific triplanar subtalar motions reinforced our subjects’ intrinsic feedback system. This leads to both higher strength output and reduced measurement error when compared to the non-FB conditions. For clinicians, strength assessment of the medio-lateral foot stabilizers is a helpful and necessary tool to identify strength deficits and to document strength adaptations during therapy and prevention programs. Reduced measurement error is essential for strength testing within the subtalar joint-specific movement plane in which the pronators and supinators of the foot are involved. Previous studies have shown beneficial medio-lateral stabilizing effects after machine-based strengthening of the pronators and supinators within their functional movement plane [5,55]. Thus, our findings on the reliability of pronator and supinator MVIC will contribute to better precision and accuracy in future strength assessments.

Assessing activity of a muscle during MVIC testing is very important for clinicians and researchers. Future research appears necessary regarding the methodology of EMG-normalization procedures for PL and TA. Although our EMG values were normalized to submaximal loads as recommended by others [35,56], the normalization procedure of heel and toe raises might be a limitation of our study. When performing these exercises, the movement of the foot-shank-complex might have had too many degrees of freedom, because, when doing this, the center of mass had to be balanced within the plantar contact area. Thus, the low reliability in EMG outcome may be a consequence of the variability in normalization. However, at least four valid normalization trials with adequate rest between these trials were recorded and averaged. While an optimized normalization procedure for triceps surae was investigated by Ball and Scurr [57], there is, to the best of our knowledge, no study scrutinizing the measurement error of isometric normalizing procedures for assessing surface EMG in PL and TA, e.g. isometric motor performance tasks, MVIC or manual testing. Hence, it remains unclear whether the high measurement errors in IEMG outcome reflect low reliability in the target motor task or the normalizing procedure or both. Therefore, more information on EMG reliability is needed for the lower leg muscles, especially when EMG is applied in pre-post strength testing to identify effects of clinical interventions.

## Conclusions

Despite the unique and difficult anatomy of the subtalar joint, pronator and supinator PTs show good within-, and acceptable to good between-day reliability in the FB conditions. The findings of our study indicate that administration of supplementary biofeedback not only enhances muscle strength output, but also reduces measurement error in subtalar joint-specific PT. As a consequence, supplementary visual biofeedback should be implemented into standardized ankle strength testing. EMG from TA is reliable during pronation within one session with and without FB. If FB is applied, TA will also show good between-session reliability. PL recordings show poor within-session reliability, especially the re-application of electrodes causes substantial measurement error between sessions during both pronator and supinator strength testing. However, researchers have to consider whether this level of error is acceptable in the context of the purpose of the planned investigation.

## Abbreviations

EMG: Electromyography
FB: Feedback
Het_R^2^: Index of heteroscedasticity
IEMG: Integrated EMG (= integral of the full-wave rectified EMG signal)
LoA: Limits of agreement
MDC: Minimum detectable change
MVIC: Maximum voluntary isometric contraction
PL: Peroneus longus muscle
PP: Peak pressure
PT: Peak torque
TA: Tibialis anterior muscle
